# Identification of Novel Compound Heterozygous Variants of the *PNPLA6* Gene in Boucher–Neuhäuser Syndrome

**DOI:** 10.3389/fgene.2022.810537

**Published:** 2022-02-07

**Authors:** Junyu He, Xin Liu, Liyi Liu, Shaohao Zeng, Shuanghong Shan, Zhihong Liao

**Affiliations:** ^1^ Department of Endocrinology, The First Affiliated Hospital, Sun Yat-sen University, Guangzhou, China; ^2^ Aegicare Technology Co., Ltd., Shenzhen, China

**Keywords:** Boucher–, Neuhäuser syndrome, *PNPLA6* gene, hypogonadotropic hypogonadism, chorioretinal dystrophy, sequencing

## Abstract

**Background:** Boucher–Neuhäuser syndrome (BNS, MIM 215470) is a rare autosomal recessive syndrome caused by mutations in the *PNPLA6* gene. Few BNS cases have been reported for functional validation at the RNA level. Herein, we report on the family of a 17-year-old girl with clinical characteristics of BNS, genetic validation, and a systematic review of *PNPLA6* variants related to BNS.

**Methods:** Clinical data and blood samples were collected from the patient and their parents, and whole-exome sequencing was performed and confirmed by Sanger sequencing. RNA-sequencing (RNA-Seq) and quantitative RT-PCR (qRT-PCR) were performed, and the three-dimensional protein structures of the variants were predicted.

**Results:** We report a 17-year-old female with progressive night blindness since the age of four, primary amenorrhea, and non-development of secondary sexual characteristics. Her impaired vision was diagnosed as retinal pigmentary degeneration of the retina. She had congenital hypogonadotropic hypogonadism (CHH) but no cerebellar ataxia at present. Two novel compound heterozygous variants (c.2241del/p.Met748TrpfsTer65 and c.2986A>G/p.Thr996Ala) of the *PNPLA6* gene (NM_006702.4) were identified by whole-exome sequencing. The former variant was carried from her healthy father and has not been reported previously. The latter was inherited from her healthy mother and was noted in a report without functional studies. The RT-PCR results showed that the mRNA expression of *PNPLA6* was lower in this patient and her father than in the control group. She was diagnosed with BNS. Both variants (c.2241del and c.2986A>G) were likely pathogenic according to the ACMG criteria. The novel variants in the *PNPLA6* gene related to Boucher–Neuhäuser syndrome were summarized in this article.

**Conclusion:** The possibility of Boucher–Neuhäuser syndrome should be considered when patients present with night blindness, impaired vision, and hypogonadotropic hypogonadism. Gene sequencing is currently the primary diagnostic method. Herein, novel compound heterozygous variants of *PNPLA6* were identified in a BNS patient, and its function was verified at the RNA level. The *PNPLA6* c.2241del variant is novel and potentially pathogenic, expanding the mutation spectrum in *PNPLA6*.

## Introduction

Congenital hypogonadotropic hypogonadism (CHH) is a rare genetic disorder characterized by insufficient or inadequate secretion of gonadotropin-releasing hormone (GnRH) (Dwyer et al., 2019). To date, 61 genes have been reported to cause CHH, including the *PNPLA6* gene (Cangiano et al., 2021). It encodes neuropathy target esterase (NTE) and is present in the cerebellum, brain, pituitary, retina, lens, testis, and kidney (Hufnagel et al., 2015). It maintains cell membrane lipid homeostasis by deacetylating phosphatidylcholine to glycerophosphorylcholine (Sogorb et al., 2016). It also maintains the homeostasis of endoplasmic reticulum phospholipids. When the homeostasis is disrupted, it will lead to delayed neurotoxicity from organophosphate poisoning (Zhu et al., 2016). In addition, a dysfunction of the NTE will lead to retinal dystrophy, intellectual disability, cerebellar ataxia, and hypogonadotropic hypogonadism, among other complications (Synofzik et al., 1993). The retinal diseases caused by *PNPLA6* can range from mild retinal pigment epitheliopathy to severe chorioretinal atrophy (DeNaro et al., 2018; Teive et al., 2018; Zheng et al., 2018).

Mutations in the *PNPLA6* gene are associated with a variety of clinical conditions, including Boucher–Neuhäuser syndrome (MIM 215470, which manifests as cerebellar ataxia, chorioretinal dystrophy, and hypogonadotropic hypogonadism) (Tarnutzer et al., 2015), Gordon Holmes syndrome (MIM 212840, which manifests as cerebellar ataxia, brisk reflexes, and hypogonadotropic hypogonadism) (Synofzik et al., 1993), Oliver–McFarlane syndrome (MIM 275400, which manifests as short stature, chorioretinal dystrophy, hypopituitarism, and intellectual disability) (Hufnagel et al., 2015), Laurence–Moon syndrome (MIM 245800) (Hufnagel et al., 2015), and spastic paraplegia type 39 (MIM 612020, which manifests as upper motor neuropathy, peripheral neuropathy, and sometimes cognitive dysfunction) (Rainier et al., 2008).

The Boucher–Neuhäuser syndrome is a disease caused by mutations in *PNPLA6*. It is a rare autosomal recessive syndrome that was first discovered by Boucher and Gibberd in two women (Boucher and Gibberd, 1969). It was characterized by spinocerebellar ataxia (SA), chorioretinal dystrophy (CD), and hypogonadotropic hypogonadism (HH) (Donaldson et al., 2020). CD frequently begins prior to the age of 50 (Synofzik et al., 1993), while HH appears in adolescence (Braslavsky et al., 2015). In contrast, SA usually occurs in early adulthood, typically between the first and third decades of life (Deik et al., 2014). However, some rare cases of late-onset ataxia have been reported in the literature, usually occurring in the third or fourth decade (Tojo et al., 1995; Ling et al., 2009; Kate et al., 2011; Deik et al., 2014).

This report is about a 17-year-old female who had progressive night blindness and sight impairment since childhood and hypogonadotropic hypogonadism since puberty. Novel compound heterozygous variants in the *PNPLA6* gene were identified by whole-exome sequencing. Functional validation by RNA-sequencing (RNA-Seq) and quantitative RT-PCR (qRT-PCR) was performed on her family. Based on genetic diagnosis and clinical features, she was diagnosed with Boucher–Neuhäuser syndrome. Furthermore, a list of the *PNPLA6* variants associated with Boucher–Neuhäuser syndrome has been compiled and reviewed.

## Methods

### Ethical Approval

The study was approved by the Ethics Committee of the First Affiliated Hospital of Sun Yat-sen University [approval number (2013) C-112]. Informed consent was obtained from the patient and her parents.

### Clinical Evaluations

A detailed ophthalmic examination, including visual acuity, visual field, fundoscopy, and optical coherence tomography (OCT), was conducted on the patient. The sex hormone, thyroid hormone, growth hormone, and IGF-1 levels were tested. Uterine ultrasound, pelvic MR, and pituitary MR were conducted on the patient, and bone age was assessed by a one-hand X-ray.

### Whole-Exome Sequencing

Whole exome sequencing was performed on the blood samples from the proband and her parents. Genomic DNA was extracted from peripheral blood samples using the QIAamp Blood Genomic DNA Extraction Kit (CAT No./ID: 51104), and the concentration and purity of DNA were detected by a NanoDrop 2000. DNA fragments were segmented into a length range of 150–300 bp using the Covaris M220 focused-ultrasonicator. Fragments of DNA were amplified after terminal repair and added to the reaction. The XGen^®^ Exome Research Panel v1.0 kit (IDT) was used to capture the amplified library, and PE150 sequencing was performed on an Illumina Novaseq 6,000.

### Sanger Sequencing

Primers for the upstream and downstream regions of the mutations were designed using Primer 5 design software based on the suspected mutation sites detected by whole-exome sequencing. The primers were synthesized by Sangon Bioengineering (Shanghai, China). The forward and reverse primers for *PNPLA6* (c.2241del) were 5′-CTG​CAG​TCT​GGG​AGC​ACA​GGA-3′ and 5′-CCT​TCT​GCA​ACA​CAC​CTA​TC CA-3'. The forward and reverse primers for PNPLA6 (c.2986A>G) were 5′-CCA​CCT​TGT​GGA​TGG​CCT​TG-3′ and 5′-TCC​TGG​AAG​ACC​CGA​TGG​ATG-3'. The genomic DNA was used as a template and amplified by Taq DNA polymerase (TAKARA). Two-way sequencing was performed with an ABI 3730XL sequencer after purification of the products. The results were analyzed with Chromas software and compared with wild-type sequences to analyze their genetic status.

### Bioinformatics Analysis

Briefly, the raw reads were preprocessed to remove low-quality reads or adaptors. Reads were compared to the default parameters of the human genome assembly hg19 (GRCh37) using the Burrows–Wheeler Aligner tool (version 0.7.17), as described previously (Li and Durbin, 2009). The SAM Genome Analysis Toolkit (GATK) was used to detect SNVs and indels (<50 bp) in the bam files, and the CNVkit was used to detect copy number variations (CNVs) (do Valle et al., 2016). Next, the Variant Effect Predictor was employed to annotate SNVs, indels, and CNVs. Several prediction tools were used to assess variations’ potential effects on protein function, including Sorting Intolerant from Tolerant (SIFT), Protein Variation Effect Analyzer (PROVEAN), MutationTaster, and Polymorphism Phenotyping version 2 (PolyPhen-2). The following rules were used to calculate homozygosity ratios with SNV data: 1) SNPs with a depth of <20-fold were excluded and 2) only SNPs with variant allele frequencies ranging from 0.2 to 0.8 were considered heterozygous.

Candidate variants associated with disease phenotypes were prioritized in this study. A series of prioritization strategies were applied to select candidate variants associated with clinical phenotypes based on the aforementioned variant annotations. The detailed steps were as follows: 1) variants with MAFs greater than 0.05 in public databases were excluded; 2) variants with no detrimental effects on any of the protein functions predicted by SIFT and Polyphen2 were excluded; and 3) variants described as benign or likely benign in ClinVar and not causing disease in HGMD were excluded. The variants that were most likely to cause disease were selected according to the clinical symptoms of the patient. Variants were classified according to the criteria of the American College of Medical Genetics (ACMG) (Richards et al., 2015).

### RNA-Sequencing

According to the GTEx Portal database, *PNPLA6* is highly expressed in the blood. Total RNA was extracted from peripheral blood samples of the patient and her parents using the PAX Gene Blood RNA Kit (Qiagen, Germany). After passing the Agilent 2100 Bioanalyzer (United States) test, 1 μg of RNA was extracted to construct an mRNA library. The poly(A)-structured mRNAs were captured and fragmented into 250 bp fragments using oligo-dT beads (Invitrogen, United States). They were then reversely transcribed into double-stranded cDNA using the KAPA mRNA HyperPrep Kit (Roche, United States) with A-tails and index connectors. They were amplified to the desired concentration before sequencing with an Illumina NovaSeq6000 (Illumina, United States). For RNA-Seq analysis, the raw reads were mapped to the GRCh38/hg19 human reference genome using HISAT2. The aligned reads were assembled and quantified using StringTie. Alternative pre-mRNA splicing events were analyzed using the Human Splicing Finder and visualized using the Integrative Genome Viewer (IGV).

### Quantitative Real-Time PCR

Total RNA was extracted from the proband, her parents, and a healthy control using a blood RNA extraction kit (QIAGEN). The extracted RNA was reverse transcribed into cDNA. qRT-PCR was then performed. The primer pair sequences were as follows: forward (5′-CAC​GTC​CAT​TGG​CTT​TCA​TC-3′) and reverse (5′-CCT​CAA​TCT​GCT​TAT​CCT​GGA​AGA-3′). GAPDH was used as a reference.

## Results

### Clinical Features

The proband, a 17-year-old female (46, XX), was presented to our hospital in July 2020 for primary amenorrhea and failure to develop secondary sexual characteristics. She presented with progressive night blindness and impaired vision since the age of four and was diagnosed as having pigmentary degeneration of the retina based on the results of visual acuity, visual field, fundoscopy, and optical coherence tomography (OCT) in an ophthalmic hospital. She had no siblings. A heterozygous variant (c.3400G>A/p. E1134K) of the *CACNA1F* gene (NM_001256789) was identified in the proband. *CACNA1F*-related disorders include Åland island eye disease (AIED, MIM 300600) and incomplete congenital stationary night blindness (ICSN), both X-linked recessive disorders. The variant of *CACNA1F* was not considered the cause of her blindness as she only carried one heterozygous variant (Mahmood et al., 2021).

The patient presented herself to a local hospital in April 2020. Laboratory tests revealed that her follicle-stimulating hormone (FSH) level was as low as 0.3 mIU/ml [reference range 3.5–12.5 mIU/ml (follicular phase)]; luteinizing hormone (LH) level was as low as 0.1 mIU/ml (reference range 2.4–12. 6 mIU/ml (follicular phase); estradiol level was as low as 18.35 pmol/L [reference range 45.4–854 pmol/L (follicular phase)]; normal prolactin level of 8.45 ng/ml (reference range 4.79–23.3 ng/ml); normal thyroid function [TSH: 3. 65 mIU/ml (reference range 0.27–4.2 mIU/ml); free triiodothyronine: 5.06 pmol/L (reference range 3.1–6.8 pmol/L); free thyroxine: 13.57 pmol/L (reference range 12–22 pmol/L); TG-Ab: 0.00 IU/ml (reference range ≤3.99 IU/ml); TPO-Ab: 5.6 IU/ml (reference range ≤9 IU/ml); TRAb: 0.9 IU/L (reference range 0–1.75 IU/L); normal GH-IGF-1 axis function [GH: 1.1 μl/L (normal range: 0–10 μl/L)]; and IGF-1: 319 ng/ml (normal range: 193–754 ng/ml). Ultrasound of the uterus showed a small uterus (10.2 × 4.5 × 8.3 mm) and a small ovary. Pelvic MR revealed an immature uterus. Pituitary MR revealed a high T2WI signal in the pituitary fossa and vacuole turcica. It showed the possibility of pituitary atrophy. She was diagnosed with congenital hypogonadotropic hypogonadism. She had no symptoms of cerebellar ataxia, brisk reflexes, trichomegaly, or intellectual disability. She was treated occasionally with Chinese herbal medicines.

The patient then came to our hospital in July 2020 for further treatment. Her vision was 0.01 in her right eye and 0.1 in her left eye, and she had severe night blindness. Her weight was 52.5 kg. Her height was 1.62 m (+1.1 SD) with a target height of 1.69 m. According to her left-hand radiograph, her bone age was 12.5 years, and she was in Tanner stage 1 due to a lack of breast and pubic hair development. Her anti-Müllerian hormone level was 1.46 ng/ml. A triptorelin (gonadotropin-releasing hormone, GnRH) stimulation test was performed. Serum LH levels were 0.13, 0.17, 0.18, 0.22, and 0.25 IU/L at 0, 30, 60, 90, and 120 min after subcutaneous injection of 0.1 mg of triptorelin. And FSH levels were 0.25, 0.25, 0.26, 0.28, and 0.33 IU/L at 0, 30, 60, 90, and 120 min. Since July 2020, she has been taking a low dose of estradiol (0.5 mg QD). On May 11, 2021, her height was 167.5 cm (+1.5 SD), her arm span was 1.67 m, and her bone age was 14 years. Her breast and pubic hair development were at Tanner stage 2 (Table 1). She also experienced leucorrhea.

**TABLE 1:** Somatometric characteristics before and after estradiol treatment.

	Before treatment	After treatment
Age (years old)	17	18
Body weight (kg)	52.5	57.5
Height (m)	1.62 (+1.1 SD)	1.675 (+1.5 SD)
Target height (m)	1.69	1.69
Arm span (m)		1.67
Bone age (years old)	12.5	14
Tanner stage	1	2

Her parents are both Han Chinese and are not consanguineous. They had normal sexual development and visual acuity. The patient’s family history was unremarkable.

### PNPLA6 Mutation Analysis

**FIGURE 1:**
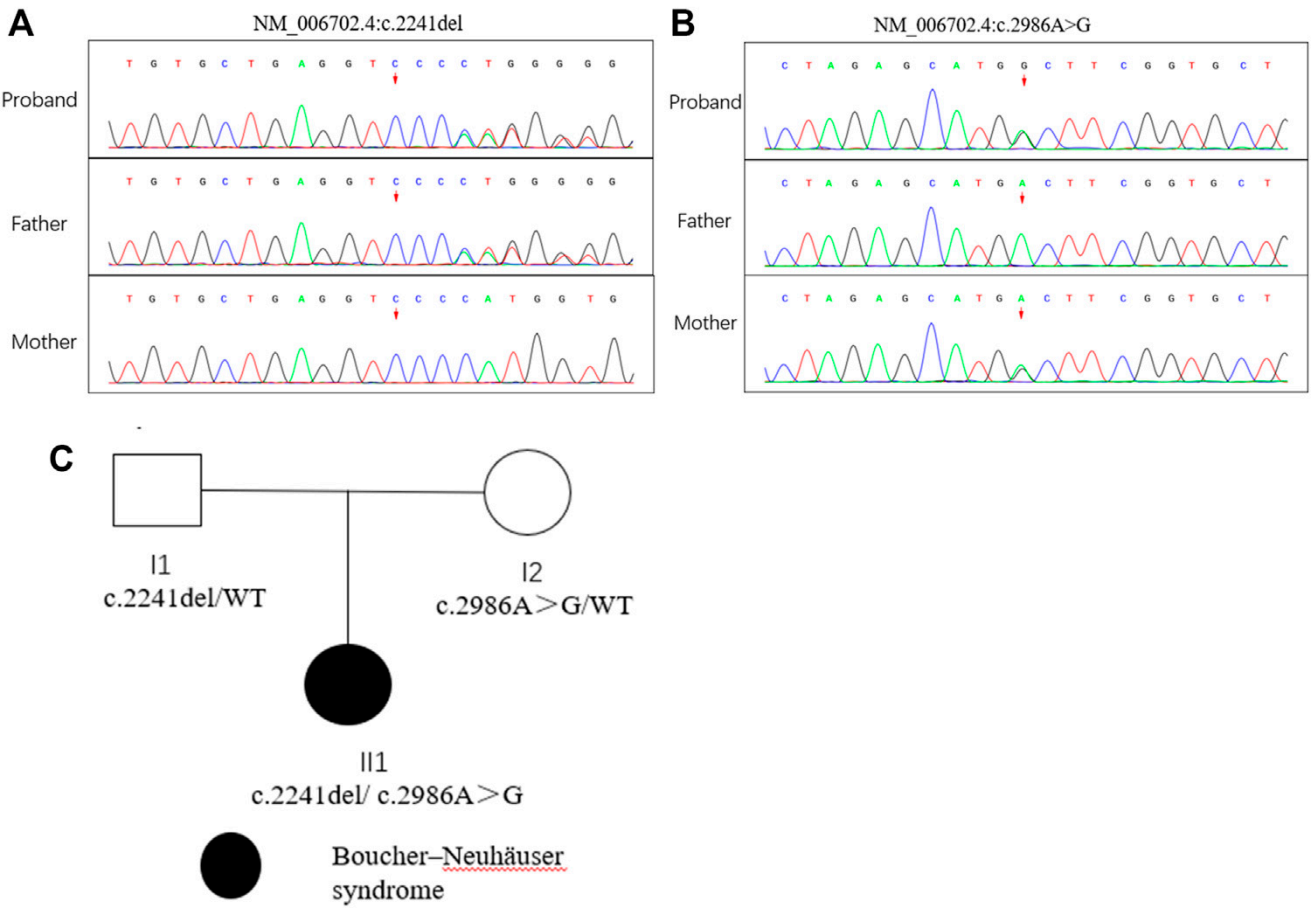
Mutation analysis of the *PNPLA6* gene and the family tree of the patient. **(A) (B)** Sanger sequencing of the *PNPLA6* gene (GenBank accession number: NM_006702.4) in the family with the variant c.2241del and c. 986A>G. **(C)** Pedigree of the family.

**FIGURE 2:**
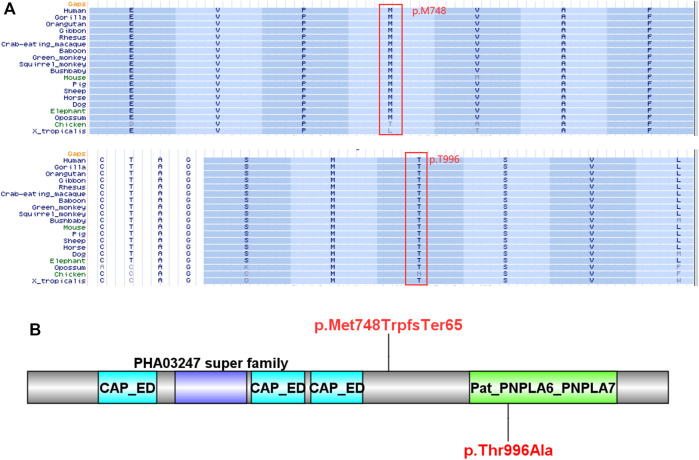
Bioinformatics analysis of the *PNPLA6* mutations. **(A)** Results of multiple amino acid alignments of *PNPLA6* orthologues in the UCSC database. **(B)** The mutation sites of the patient in the protein domain of *PNPLA6*.

**FIGURE 3:**
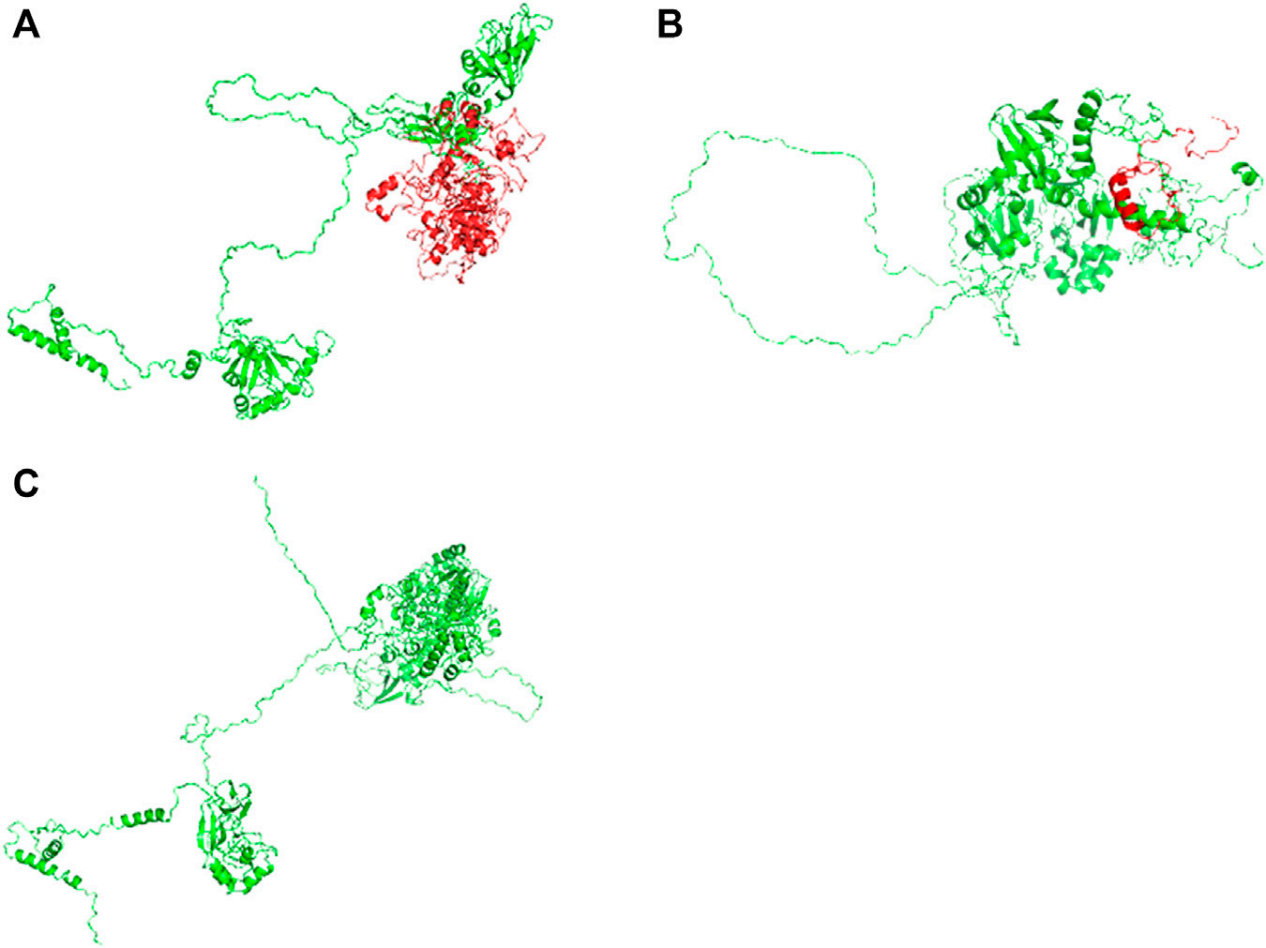
Three-dimensional structures of the wild-type and mutant proteins: **(A)** Wild-type protein; **(B)** mutant proteins of the variant c.2241del; **(C)** mutant protein of the variant c.2986A>G.

Compound heterozygous variants (c.2241del/p. Met748TrpfsTer65 and c.2986A>G/p.Thr996Ala) were identified in the *PNPLA6* gene (NM_006702.4) by whole-exome sequencing in the proband and validated by Sanger sequencing (Figure 1). The coverage of the target genes was 99.6% (above 10-fold), 99.47% (above 20-fold), and 98.99% (above 30-fold). The average sequencing depth of the WES data was 129. The c.2241del variant was from her father, an unreported variant. The c.2986A>G variant was from her mother and has been reported previously (Wu et al., 2021). The former caused deletion of nucleotide 2,241 of the *PNPLA6* gene (c.2241del), resulting in the substitution of methionine by tryptophan in codon 748 of the *PNPLA6* protein and termination at downstream codon 65 (P. Met748TrpfsTer65), which is a frameshift mutation. The minimum allelic frequency of this variant has not been recorded in the Genome Aggregation Database (gnomAD) and has not been reported in the ClinVar or HGMD databases. The c.2986A>G variant caused the substitution of A by G in nucleotide 2,986 of the *PNPLA6* gene, resulting in *PNPLA6* protein 996 threonine in codon to alanine (p. Thr996Ala), which is a missense mutation. The minimum allele frequency of this variant in gnomAD is 0.00027. The c.2986A>G variant has 0 homozygosities in gnomAD. This mutation was not reported in either the ClinVar or HGMD databases. We found that amino acids from p.M748 and p. T996 are highly conserved in many species, implying a higher probability of pathogenicity (Figure 2A). Moreover, several *in silico* programs were used to assess the pathogenicity of the variants. The c.2241del variant is a frameshift mutation, and only a few programs have been able to evaluate this variant. MutationTaster was a tool often used. The assessment results showed that the c.2986A>G variant might be deleterious. The c.2986A>G variant was predicted to be deleterious or possibly damaging by MutationTaster, PolyPhen-2, LRT, PROVEAN, Eigen, M_CAP, FATHMM_MKL, GenoCanyon, and fitCons. However, other programs predicted it to be benign or tolerable (Supplementary Table S1). In addition, the three-dimensional structure of the mutant protein was different from the wild-type protein (Figure 3). Apart from *PNPLA6*, we did not find any additional concerning variants in the 61 genes reported in HH. In addition, the karyotype of the proband was 46, XX.

### RNA-Sequencing and Quantitative Real-Time PCR

**FIGURE 4:**
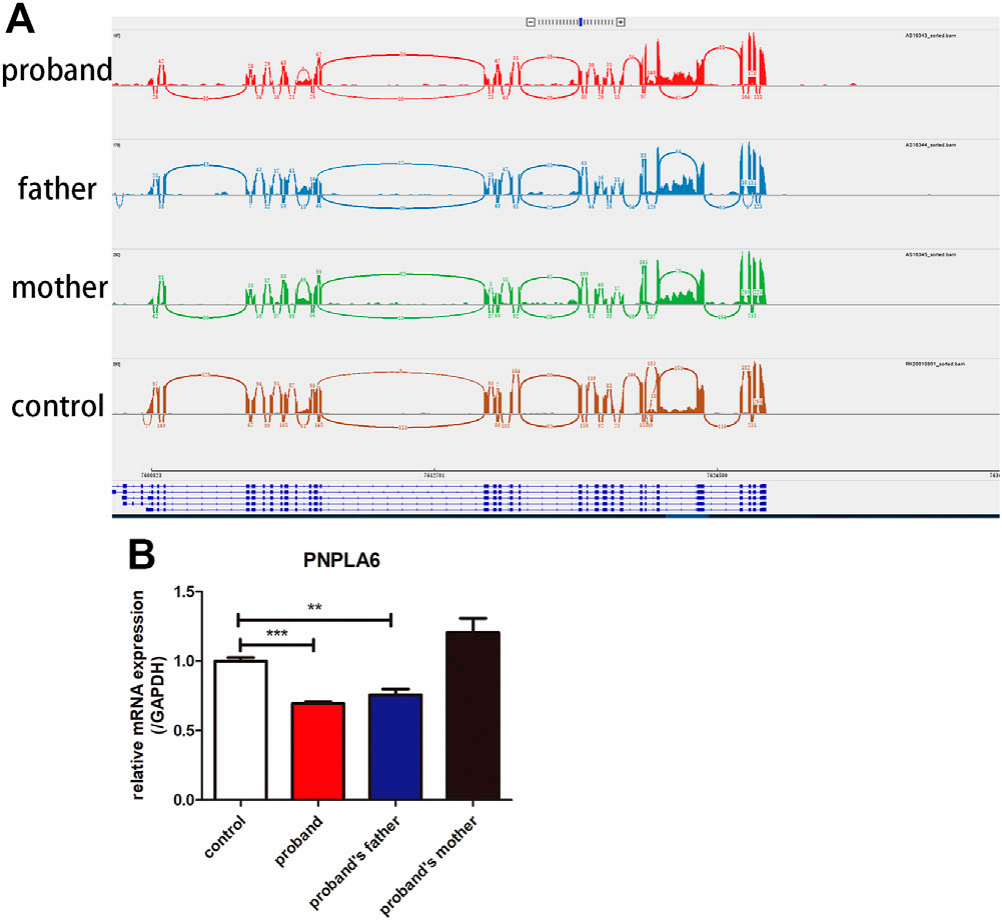
Results of RNA-Seq and qRT-PCR of the proband and her parents. **(A)** RNA-Seq showed that no abnormal splicing was detected in the families; **(B)** RT-PCR showed that the mRNA expression of *PNPLA6* was lower in the proband and her father compared to the control. **p < 0.01, ***p < 0.005.

There was no abnormal splicing in these families by RNA-Seq compared to the healthy control (Figure 4A). The qRT-PCR results showed decreased RNA expression of *PNPLA6* in the proband and her father compared to the healthy control (Figure 4B). No significant differences were found between the proband’s mother and the control group. This suggests that the compound heterozygous and frameshift variants of c.2241del result in decreased mRNA expression of *PNPLA6*, which confirms that this frameshift mutation is pathogenic. The c.2241del variant was predicted to be likely pathogenic (LP = PVS1 + PM2), and the c.2986A>G variant was also likely pathogenic (LP = PM1 + PM2 + PM3 + PM5) according to the ACMG guidelines (Table 2).

**TABLE 2:** Diagnosis of variants from ACMG criteria.

Variant of *PNPLA6*	Diagnostic criteria from ACMG
NM_006702.4:c.2241del	1. The nonfunctional variants (nonsense mutation, frameshift mutation, splicing mutation of classical ±1 or 2, start codon variation, deletion of one or more exons) when the pathogenic mechanism of the disease is loss of function (LOF) (PVS1)
	2. The variants not found in normal individuals (or very low-frequency loci in recessive genetic disorders) in ESP, 1,000, EXAC databases (PM2)
NM_006702.4:c.2986G>A	1. The variants with a mutational hotspot in the protein domain (PM1)
	2. The variants not found in normal individuals (or extremely low-frequency loci in recessive genetic diseases) in ESP, 1,000, EXAC databases (PM2)
	3. The variants in trans with a pathogenic variant (PM3)
	4. The variants previously reported (PM5)

## Discussion

Boucher–Neuhäuser syndrome (BNS) is a rare autosomal recessive disorder that often develops at a young age and is caused by mutations in the *PNPLA6* gene. It is characterized by spinocerebellar ataxia, chorioretinal dystrophy, and hypogonadotropic hypogonadism. Chorioretinal dystrophy often presents with impaired vision and night blindness and can be diagnosed by fundoscopy and optic coherence tomography (OCT). In OCT, retinal thinning loss of layered retinal structures, choriocapillaris, and choroidal vessels can be detected (Synofzik et al., 1993).

The patient had retinitis pigmentosa and idiopathic hypogonadotropic hypogonadism due to visual impairment during childhood and hypoplastic secondary sexual characteristics during puberty. Compound heterozygous variants (c.2241del/p.Met748TrpfsTer65 and c.2986A>G/p.Thr996Ala) were identified in the *PNPLA6* gene by whole-exome sequencing. The former variant has not been reported previously. To our knowledge, there are three syndromes caused by mutations in the *PNPLA6* gene that manifest as chorioretinal dystrophy and hypogonadotropic hypogonadism. They are Boucher–Neuhäuser syndrome, Oliver–McFarlane syndrome, and Laurence–Moon syndrome. The Boucher–Neuhäuser syndrome is distinguished from the Oliver–McFarlane syndrome by the absence of trichomegaly, short stature, and intellectual disability (Hufnagel et al., 2015). It can also be distinguished from the Laurence–Moon syndrome by the presence of childhood-onset ataxia, peripheral neuropathy, and spastic paraplegia (Hufnagel et al., 2015). This patient had no signs of childhood-onset ataxia, peripheral neuropathy, or spastic paraplegia.

Moreover, the patient did not exhibit trichomegaly, short stature, or mental retardation. Therefore, based on her clinical features and genetic findings, she was diagnosed with Boucher–Neuhäuser syndrome. It has been reported that a patient with BNS does not develop cerebellar ataxia, such as gait imbalance and veering to either side, until age 50. This is referred to as late-onset ataxia (Deik et al., 2014). Another patient with Boucher–Neuhäuser syndrome developed gait imbalance at 50 years of age (Deik et al., 2014). In addition, a patient from Japan noted gait instability and speech difficulties at the age of 35. This proband does not currently have cerebellar ataxia, which may be a possibility for late-onset ataxia. Therefore, she should be observed for gait ataxia, scissor gait, or spasticity in the future.

The *PNPLA6* gene is on chromosome 19p13.2, encoding the neurogenic target esterase (NTE). It has five different transcripts, with the longest one encoding a protein of 1,375 amino acids (Xu et al., 2016). The conserved structural domain of NTE contains three effector domains of CAP family transcription factors and a patatin-like phospholipase structural domain-containing protein 6 and protein 7 (Marchler-Bauer et al., 2017). Protein 6 (*PNPLA6*) and protein 7 (*PNPLA7*) containing the patatin-like phospholipase structural domain are 60% identical. *PNPLA6* is commonly referred to as a neuropathy-targeted esterase (NTE), which shows phospholipase activity towards lysophosphatidylcholine (LPC) and phosphatidylcholine (PC) (Kienesberger et al., 2009). PNPLA7 is an insulin-regulated phospholipase, homologous to neuropathy-targeted esterase (NTE or *PNPLA6*), also known as NTE-related esterase (NRE). The enzyme NRE hydrolyzes sn-1 esters in lysophosphatidylcholine and lysophosphatidic acid but lacks lipase activity for substrates such as triacylglycerols, cholesteryl esters, retinyl esters, phosphatidylcholine, or monoacylglycerol (Kienesberger et al., 2008, 2009). In contrast, the effector domains of CAP family transcription factors include CAP (cAMP receptor protein (CRP), CooA (heme-containing CO sensor), and FNR (fumarate and nitrate reduction). This will lead to conformational changes and activate transcription (Chan, 2000; Liu et al., 2020).

**FIGURE 5:**
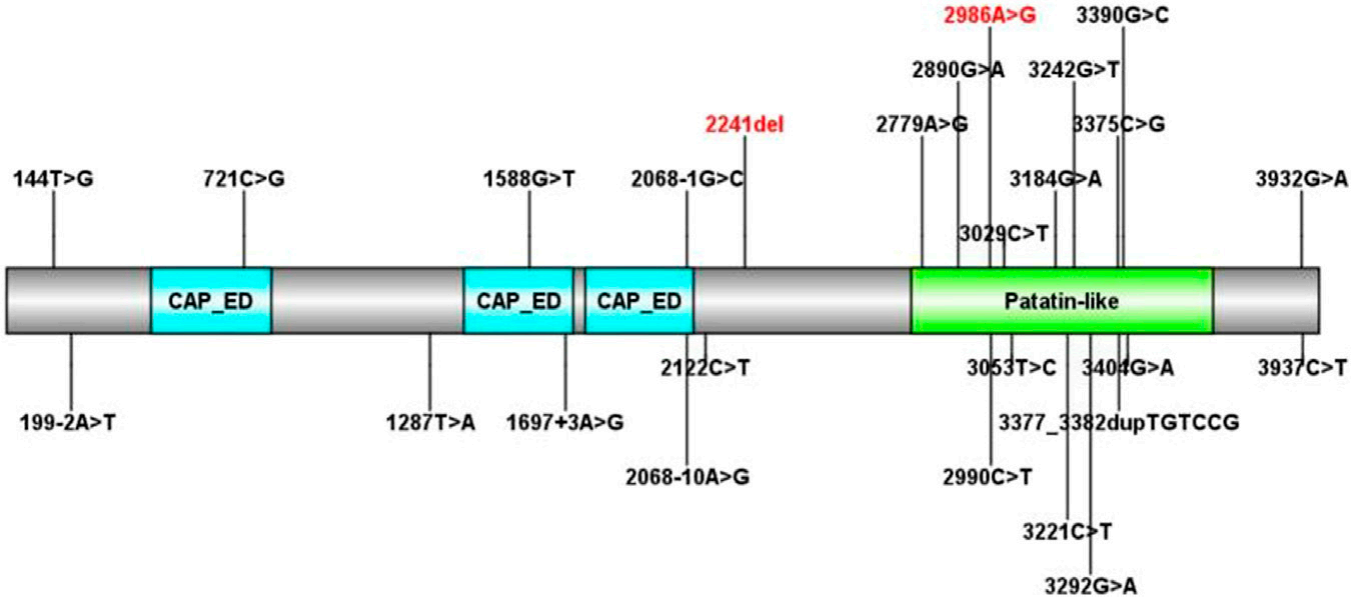
All *PNPLA6* gene mutations in Boucher–Neuhäuser syndrome (the variants marked in red are from this report).

To date, there are 83 pathogenic variants of the *PNPLA6* gene according to the HGMD database, previous literature, and this report. They are summarized in Table 3 (Wu et al., 2021; Liu et al., 2020; Emekli et al., 2020; Sen et al., 2020; Makuloluwa et al., 2020). Of these, 25 variants (30.1%) were associated with Boucher–Neuhäuser syndrome, 13 variants (15.7%) with Oliver–McFarlane or Laurence–Moon syndrome, 8 (9.6%) variants with Gordon Holmes syndrome, and the rest with other diseases. Most of the variants related to Boucher–Neuhäuser syndrome are located in the patatin-like phospholipase structural domain, which determines the esterase activity of NTE, as shown in Figure 5. Most of the variants fall into the C-terminal phospholipid esterase structural domain, which will inhibit the catalytic activity of NTE (Synofzik et al., 2014). Several researchers have found that variants at the N-terminal end of the patatin-like phospholipase structural domain may be associated with spastic paraplegia and spastic ataxia. In contrast, variants at the C-terminal end of the patatin-like phospholipase structural domain may be associated with cerebellar ataxia and hypogonadism (Synofzik et al., 2014). It was also found that chorioretinal dystrophy is closely related to variants of the patatin-like phospholipase domain (Wu et al., 2021). In this report, the novel variant of c.2241del is a frameshift mutation and is not in a structurally important domain. However, this mutation leads to premature truncation of the mRNA at an early length. It will cause a highly unstable mRNA that could eventually undergo nonsense-mediated decay, leading to decreased expression levels of *PNPLA6* in the proband and her father. In contrast, the c.2986A>G variant is a missense mutation previously reported in a 25-year-old female BNS patient (Wu et al., 2021). This patient carried an insertional mutation and a missense mutation and presented with chorioretinopathy and hypogonadotropic hypogonadism without neurological symptoms, which was similar to our patient. However, she was short in stature (below the 5th percentile), different from our report. The c.2986A>G variant is located in the patatin-like phospholipase domain and may disrupt the deacetylation of phosphatidylcholine to glycerophosphatidylcholine. Moreover, this would disrupt the homeostasis of cell membrane lipids (Hufnagel et al., 2015). Although no abnormal splicing was found in the family, mRNA expression was lower in the patient and her father than the control by RT-PCR, suggesting that the compound heterogeneous variant and frameshift mutation of c.2241del resulted in decreased mRNA expression of *PNPLA6*. According to the ACMG criteria, the c.2241del variant was predicted to be likely pathogenic (LP = PVS1 + PM2), and the c.2986A>G variant was also likely pathogenic (LP = PM1 + PM2 + PM3 + PM5). Both variants lead to protein translation, and the former variant even leads to premature translation termination, which disrupts the function of NTE and causes many clinical symptoms in the patient.

**TABLE 3:** Pathogenic variants in *PNPLA6* (NM_006702.4) that have been reported.

Clinical phenotypes	Variant in *PNPLA6*
Boucher–Neuhäuser syndrome (chorioretinal dystrophy, hypogonadotropic hypogonadism, and cerebellar ataxia)	3053T>C, 3377_3382dupTGTCCG, 1588G>T, 3932G>A, 2779A>G, 144T>G, 2068-1G>C, 3184G>A, 199-2A>T, 3375C>G, 3242G>T, 3029C>T, 3404G>A, 2990C>T, 2890G>A, 3221C>T, 3937C>T, 721C>G, 3390G>C, 1287T>A, 1,697+3A>G, 3292G>A, 2068-10A>G, 2122C>T, 2986A>G^a^, 2241del^a^
Gordon Holmes syndrome (cerebellar ataxia, brisk reflexes, and hypogonadotropic hypogonadism)	3931C>T, 3380C>G, 3295C>T, 3940C>G, 2297T>C, 1126dupG, 2,494_2495insTGTGGGCCTGGGG, 3387G>A
Oliver–McFarlane/Laurence–Moon syndrome (Oliver–McFarlane syndrome: short stature, chorioretinal dystrophy, hypopituitarism, and intellectual disability	1829+2T>G, 2032G>C, 3152G>A, insertion 9904bp incl. ex. 14–20, 3382G>A, 3241G>A, 3476C>A, 3500T>C, 1491G>T, 3367G>A, 3702+1G>A, 3190G>A, c.3403C>T, c.830G>C
Laurence–Moon syndrome: chorioretinal dystrophy and hypogonadotropic hypogonadism, childhood onset of ataxia, peripheral neuropathy, and spastic paraplegia)	
Retinal degeneration/retinitis pigmentosa	1094dupC, 932C>T, 1427T>C, 3241G>C, 1972C>T, 3229G>A, 2619G>A, 3178C>T, 3190G>A, 3403C>T
Motor neuron disease	3034A>G, 2,944_2947dupAGCC, 2669G>A
Cerebellar ataxia/sporadic ataxia	1339C>A, 1340C>T, 2,779_2780insAA, 3847G>A, 1713G>T, 3785A>T, 3598C>G, 3155T>G
Amyotrophic lateral sclerosis	2914G>A, 532G>T
Hypogonadotropic hypogonadism	1742C>G
Spastic paraplegia type 39	2378G>C, 643G>A, 2245G>A, 3441C>G, 2375G>A, 3889C>T, 3190G>A
ARHSP (autosomal recessive hereditary spastic paraplegia)	1,672_1674delCGGinsTA, deletion ex. 17–18
Autism spectrum disorder	2423C>T
Neurology pediatric	577G>T
Spastic ataxia	2489G>A, 796C>T
Spasticity	2639G>C

a Indicating the variants that have been reported in the present study.

There were many *in vivo* and *in vitro* studies on the pathogenic mechanism of the *PNPLA6* gene. Several studies have shown that *PNPLA6* is expressed in the inner segment plasma membrane of photoreceptors and its mutation in *Drosophila* leads to the death of photoreceptor cells, which may be caused by abnormal phospholipid metabolism (Kmoch et al., 2015). This could cause retinal degeneration due to *PNPLA6* mutations. *PNPLA6* was detected in brain neurons, particularly Purkinje cells in the cerebellum, cerebral cortex, and hippocampus (Moser et al., 2000). It was also expressed in Schwann cells of the mouse sciatic nerve, most prominently in non-myelinated Schwann cells. It was involved in developing non-myelinating Schwann cells and the demyelination of neurons after neuronal injury (McFerrin et al., 2017). This may be the reason for the development of neuropathy syndrome in *PNPLA6* mutations. *In vivo* and *in vitro* studies have shown that *PNPLA6* is associated with embryonic development. The embryos from NTE null mice do not survive, and the knockout of the *PNPLA6* gene in mouse embryonic stem cells alters the nervous and vascular systems (Pamies et al., 2014). Topaloglu et al. discovered that *PNPLA6* gene mutations disrupt phospholipid homeostasis. It reduced the gonadotropin response by decreasing extracellular leakage of gonadotropin-releasing hormone (GnRH) stimulation. However, it did not affect the GnRH signaling pathway or the synthesis of the luteinizing hormone (LH) subunit. This also explains why extended stimulation did not produce a significant LH response, contributing to hypogonadotropic hypogonadism (Topaloglu et al., 2014).

In this case, the patient had compound heterozygous variants of the *PNPLA6* gene (c.2241del/p. Met748TrpfsTer65 and c.2986A>G/p.Thr996Ala). This novel mutation alters the protein structure of the NTE encoded by *PNPLA6*. We hypothesized that it would lead to reduced NTE hydrolase activity and cause abnormalities in phospholipid metabolism, resulting in impaired vision, non-developed secondary sexual characteristics, primary amenorrhea, and suspected cerebellar ataxia in the future.

We identified a novel variant of c.2241del in the *PNPLA6* gene and validated it by RNA-Seq and qRT-PCR. The 2986A>G variant has been reported previously but has not been validated at the RNA level in the previous study (Wu et al., 2021). There were few reports on using RNA-Seq and qRT-PCR for BNS’s functional validation. We demonstrated that compound heterozygous variants in the patient led to decreased mRNA expression of *PNPLA6*, although no abnormal splicing was found. We know that the c.2241del variant is not in a crucial structural domain, and its function is still unclear, requiring further basic studies. Furthermore, the obvious association of various clinical manifestations with *PNPLA6* gene variants and the pathogenic mechanism of the *PNPLA6* gene also needs further study. Variants should be investigated in the future.

In conclusion, novel compound heterozygous variants in the *PNPLA6* gene were identified through whole-exome sequencing and validated by RNA-Seq and qRT-PCR in a patient with retinitis pigmentosa and hypogonadotropic hypogonadism. She was diagnosed with Boucher–Neuhäuser syndrome. Further studies are needed to investigate the relationship between the different variants and the clinical phenotype, as well as the pathogenic mechanisms of the variants.

## Data Availability

The datasets presented in this study can be found in online repositories. The names of the repository/repositories and accession number(s) can be found below: CNGB (https://db.cngb.org/cnsa/); accession number: CNP0002563; http://db.cngb.org/cnsa/project/CNP0002563/reviewlink/.
