# Supplementary material for: Identification of Novel Compound Heterozygous Variants of the PNPLA6 Gene in Boucher–Neuhäuser Syndrome
Source: Front Genet. 2022 Feb 7;13:810537. doi: 10.3389/fgene.2022.810537 (PMC8859865; doi:10.3389/fgene.2022.810537)
Supplement: Supplementary file 1 [file Table1.DOCX]

**Supplementary table 1 The pathogenicity prediction results of variant (NM_006702.4:c.2986G＞A) by silico programs.**

|  | score | prediction |
| --- | --- | --- |
| SIFT | 0.134 | Tolerable |
| Polyphen2_HDIV | 0.917 | Possibly_damaging |
| Polyphen2_HVAR | 0.588 | Possibly_damaging |
| LRT | 0 | Deleteriou |
| MutationTaster | 1 | Disease_causing |
| MutationAssessor | 1.07 | Low |
| FATHMM | -1.23 | Tolerable |
| PROVEAN | -3.42 | Damaging |
| VEST3 | 0.196 | Tolerable |
| MetaSVM | -0.369 | Tolerable |
| MetaLR | 0.385 | Tolerable |
| M_CAP | 0.114 | Damaging |
| CADD | 14.74 | Tolerable |
| DANN | 0.987 | Tolerable |
| FATHMM_MKL | 0.998 | Damaging |
| Eigen | 0.311 | Damaging |
| GenoCanyon | 1 | Damaging |
| fitCons | 0.707 | Damaging |
| GERP | 4.98 | Conserved |
| phyloP | 9.301 | Conserved |
| phastCons | 1 | Conserved |
| SiPhy | 12.649 | Conserved |
| ReVe | 0.525 | Tolerable |
| ClinPred | 0.36187708 | benign |
